# Porcine Epidemic Diarrhea Virus Envelope Protein Blocks SLA-DR Expression in Barrow-Derived Dendritic Cells by Inhibiting Promoters Activation

**DOI:** 10.3389/fimmu.2021.741425

**Published:** 2021-11-10

**Authors:** Jie Wang, Yajing Wang, Bing Liu, Yunwei He, Zhiwei Li, Qin Zhao, Yuchen Nan, Chunyan Wu

**Affiliations:** ^1^ Department of Preventive Veterinary Medicine, College of Veterinary Medicine, Northwest Agriculture & Forestry (A&F) University, Yangling, China; ^2^ Scientific Observing and Experimental Station of Veterinary Pharmacology and Veterinary Biotechnology, Ministry of Agriculture, Yangling, China

**Keywords:** porcine epidemic diarrhea virus, PEDV-envelope protein, SLA-DR, MHC-II, subunit vaccine, adaptive immune response 3

## Abstract

Porcine epidemic diarrhea (PED) is an acute, highly contagious intestinal swine disease caused by porcine epidemic diarrhea virus (PEDV). In addition to known PEDV infection targets (villous small intestinal epithelial cells), recent reports suggest that dendritic cells (DCs) may also be targeted by PEDV *in vivo*. Thus, in this study we used bone marrow-derived dendritic cells (BM-DCs) as an *in vitro* model of antigen-presenting cells (APCs). Our results revealed that PEDV replicated in BM-DCs and that PEDV infection of cells inhibited expression of swine leukocyte antigen II DR (SLA-DR), a key MHC-II molecule involved in antigen presentation and initiation of CD4^+^ T cell activation. Notably, SLA-DR inhibition in BM-DCs did not require PEDV replication, suggesting that PEDV structural proteins participated in SLA-DR transcriptional inhibition. Moreover, reporter assay-based screening indicated that PEDV envelope protein blocked activation of SLA-DRα and β promoters, as did PEDV-ORF3 protein when present during PEDV replication. Meanwhile, treatment of PEDV-infected BM-DCs with MG132, a ubiquitin-proteasome degradation pathway inhibitor, did not restore SLA-DR protein levels. Additionally, PEDV infection of BM-DCs did not alter SLA-DR ubiquitination status, suggesting that PEDV infection did not affect SLA-DR degradation. Furthermore, additions of PEDV structural proteins to HEK-293T-SLA-DR stably transfected cells had no effect on SLA-DR protein levels, indicating that PEDV-mediated inhibition of SLA-DR expression acted mainly at the transcriptional level, not at the protein level. These results provide novel insights into PEDV pathogenic mechanisms and viral-host interactions.

## Introduction

Porcine epidemic diarrhea (PED) is an acute, highly contagious intestinal disease in swine caused by porcine epidemic diarrhea virus (PEDV). PEDV is an enveloped, positive-sense, single-stranded RNA virus belonging to the genus *Alphacoronavirus* within the family *Coronaviridae* (1) that forms pleomorphic virions with diameters ranging from 95 to 190 nm. Pigs of all ages can be infected by PEDV, with infected animals exhibiting symptoms that can vary in type and severity which include vomiting, severe watery diarrhea, and dehydration. PEDV mortality in neonatal and sucking piglets can approach 100%, resulting in huge economic losses for the swine industry worldwide (2).

The genome of PEDV is about 28 kb in length and contains at least six open reading frames (ORFs) (3). ORF1a and ORF1ab are viral replicase proteins that are translated directly from the PEDV genome as a polyprotein precursor. This precursor is subsequently cleaved by viral protease into 16 nonstructural proteins (nsps) that play separate functional roles during PEDV genomic RNA replication (2). The other PEDV-ORFs encode an accessory protein ORF3 and four structural proteins, namely, spike (S), envelope (E), membrane (M), and nucleocapsid (N) proteins (2). The spike protein is critically important for mediating virus interactions with permissive host cell receptors to effect viral binding and entry, while also serving as an antigen for eliciting host anti-PEDV neutralizing antibody responses (4). Each spike protein molecule contains two structurally distinct regions, the S1 region (aa 1 to 726 based on PEDV prototype strain CV777 sequence) and the S2 region (aa 727–1386 based on the CV777 sequence) (5). The N-terminal portion of the S1 region contains the receptor-binding domain, while the C-terminal portion of the S2 region contains the domain responsible for virus-host membrane fusion (5). Generally, it is believed that aminopeptidase N (APN) is the cellular receptor utilized by PEDV and other *Alphacoronavirus* members to mediate target cell infection (6–8).

PEDV infection *in vivo* mainly targets the villous epithelia of the small intestine, resulting in blunting of affected villi and disruption of mucosal barrier integrity (9). Although the exact host cellular targets of PEDV are unknown, candidates include enterocytes, goblet cells, Paneth cells, microfold cells, tuft cells, and stem cells (10). Nevertheless, the small intestine, which is predominantly comprised of villi and crypts (10), harbors PEDV antigen within crypt-villus interfaces during early-stage acute PEDV infection of nursing pigs, implicating immature enterocytes as major target cells of initial PEDV infection (11). Subsequently, PEDV antigen-positive foci then expand to include the upper epithelium then the entire villous epithelium area encompassing the jejunum to the ileum (11).

Dendritic cells (DCs), which pervade the tissues beneath the intestinal epithelium, can also act as a portal for virus invasion (12) and are the most potent antigen-presenting cells within the host. Ultimately, DCs are key initiators of adaptive immune responses, while also regulating the balance between tolerance and immunity within the intestinal mucosa (12). It is notable that PEDV antigens are frequently detected in intestinal crypt cells, as well as in antigen presenting cells (APCs) such as macrophages within the lamina propria or Peyer’s patches (2). Indeed, one *in vitro* study demonstrated that monocyte-derived dendritic cells (MoDCs) were susceptible to PEDV infection (13). However, results of another report suggested that PEDV failed to replicate within porcine MoDCs but did activate transcription of type I interferons and chemokine genes (14). Therefore, a controversy exists with regard to the ability of PEDV to infect APCs such as DCs. Nevertheless, if PEDV infection of DCs does occur, the biological significance of such infection and the roles of DCs in PEDV pathogenesis are still unclear. Normally when DCs function as APCs, they capture internal/exogenous antigens, process them to generate immune peptides, then present the immunopeptides on cell surfaces in association with MHC-I or II molecules. Presentation of immune peptides by MHC molecules then initiates activation of antigen-specific T cells to evoke humoral and cellular immune responses against pathogens (15, 16), a process known to involve DCs located within the intestinal lamina propria (17).

In this study, we employed bone marrow-derived dendritic cells (BM-DCs) to serve as an *in vitro* APC model. Our results revealed that PEDV could replicate in BM-DCs based on detection of PEDV-N protein in infected cells using immunofluorescence and Western blot assays, while UV-inactivated virus failed to infect cells. Notably, our data suggested that PEDV infection of BM-DCs inhibited expression of swine leukocyte antigen II DR chain (SLA-DR), a key member of the MHC-II family of molecules that participate in processes that include antigen presentation and initiation of CD4^+^ T cell activation. By revealing the molecular mechanism associated with down-regulation of SLA-DR expression in PEDV-mediated antigen-presenting cells (DC), results of this study should enhance our understanding of mechanisms whereby PEDV evades the host acquired immune response.

## Materials and Methods

### Cells, Viruses, Chemicals, and Plasmids

Vero cells and HEK-293T cells were maintained in Dulbecco’s Modified Eagle Medium (DMEM; Biological Industries, Israel) supplemented with 10% fetal bovine serum (FBS) (Biological Industries). Primary bone marrow cells were collected from femur bones of 4 weeks old piglets (male, out-breeded) obtained from PRRSV- and PEDV- free pig farm (conventional) nearby Yangling, Shaanxi, China. The piglets were subjected to screening of CSFV, PRRSV, PEDV, PCV2 and ASFV along with corresponding antibodies by government authorized agency (Shaanxi Innolever Biotechnology Co., Ltd., Yangling, Shaanxi, China). The porcine alveolar macrophages (PAMs) were collected from the same piglets as well. Porcine bone marrow-derived dendritic cells (BM-DCs) were obtained as previously described by stimulating bone marrow cells with 40 ng/mL of porcine granulocyte-macrophage colony-stimulating factor (GenScript, Nanjing, Jiangsu, China) for six consecutive days (18). Only suspended cells with typical dendritic cell morphology as previous described (19) were collected on day seven for use in experiments. Virus strains used here included PEDV Vero cell-adapted strain KB2013-p120 derived from its parental strain KB2013-4 (GenBank accession number: KX580953.1), virulent PEDV strain CH/hubei/2016 (GenBank accession number: KY496315.1), and a field PEDV isolate SXYL-21 (full sequence unavailable and sequencing is ongoing). PEDV strain stocks were propagated and titrated in Vero cells as previously described (20).

All PEDV structural proteins and ORF3 were cloned from cDNA of KB2013-p120 and were ligated to the pCAGEN vector containing DNA encoding a MYC-tag as previously described (21). The cDNA sequences of SLA-DRα and β were cloned from total PAMs cDNA as previously described (18) then cDNAs were ligated to pLVX-zsGreen (for SLA-DRα) and pLVX-mCherry (for SLA-DRβ) lentiviral vectors, respectively. SLA-DRα and β constructs were introduced into HEK-293T cells by lentivirus-mediated transduction. Cells positive for both zsGreen and mCherry (double-positive cells) were sorted using a FACS Aria™ III cell sorter (BD Biosciences, San Jose, CA, USA) and further subjected to subcloning using limited dilution. Successful expression and assembly of SLA-DR αβ heterodimers was confirmed using a homemade mouse monoclonal antibody (Clone No. MY533) that specifically recognizes SLA-DR αβ heterodimers for use in pull-down assays to detect assembled SLA-DR heterodimers *via* immunoprecipitation. Immunoprecipitated proteins subjected to Western blot analysis were probed with SLA-DRα-specific Mab. Sequences of primers used for vector construction are listed in **Table 1**. Transfections of indicated plasmids into cells of the HEK-293T-SLA-DR stable cell line were conducted using FuGENE^®^ HD Transfection Reagent (Promega) according to the manufacturer’s instructions.

**Table 1:** List of primers using for plasmids construction.

Primers	Sequence (5’-3’)	Description
PEDV-S2-F	ttcctcgagatgagtattaggacag	Cloning of S2
PEDV-S2-R	gtgcggccgcTCACAGATCCTCTTCAGAGATGAGTTTCTGCTCctgcacgtggaccttt	
PEDV-M-F	tcctcgagatgtctaacggttcta	Cloning of M
PEDV-M-R	gtgcggccgcTCACAGATCCTCTTCAGAGATGAGTTTCTGCTCgactaaatgaagcact	
PEDV-N-F	tcctcgagatggcttctgtcagtt	Cloning of N
PEDV-N-R	gtgcggccgcTCACAGATCCTCTTCAGAGATGAGTTTCTGCTCatttcctgtgtcgaag	
PEDV-E-F	tcctcgagatgctacaattagtga	Cloning of E
PEDV-E-R	gtgcggccgcTCACAGATCCTCTTCAGAGATGAGTTTCTGCTCtacgtcaataacagta	
PEDV-ORF3-F	tcctcgagatgtttcttggactttttca	Cloning of ORF3
PEDV-ORF3-R	tgcggccgcTCACAGATCCTCTTCAGAGATGAGTTTCTGCTCttcactaattgtagcat	
SLA-DRα-promoter-F	GCCTGTCGACGCGTAGAATTCACTCATCTGGCCCGTT	Cloning of SLA-DRα-promoter
SLA-DRα-promoter-R	GTGTCAGAAGAATCAAGCTTTGACTGATTAAAATTT	
SLA- DRβ-promoter-F	GCCTGTCGACGCGTAGAATTCGTCTGTTTCATACACC	Cloning of SLA-DRβ-promoter
SLA-DRβ-promoter-R	GTGTCAGAAGAATCAAGCTTGAGGCACCTGAATTGA	

### Ethics Statement and Animal Studies

Six-week-old female BALB/c mice were obtained from Dashuo Biotech (Chengdu, Sichuan, China). All animal experiments were conducted following protocols recommended by The ARRIVE guidelines which had been reviewed and approved by the Animal Welfare Committee of Northwest A&F University (Approve No. CVM-2019-NY11). All mice were monitored on a daily basis for any clinical signs. Efforts were made to minimize suffering of mice and euthanasia was performed as humanely as possible according our protocol.

Preparation of mouse sera against PEDV-N protein was conducted as previously described (18, 22). Briefly, cDNA encoding PEDV-N was amplified from total PEDV cDNA then was ligated to the pET-28a vector (23). The resulting pET-28a vector encoding PEDV-N protein as mentioned above was transformed into Escherichia coli strain BL21 (DE3) and cultured in LB medium at 37°C until induction of protein expression was initiated by addition of 0.5 mM isopropyl β-D-thiogalactoside (IPTG) followed by incubation at 25°C for 16 hours. After IPTG induction, bacterial cells were collected and resuspended in cell lysis buffer then were sonicated as previously described (18). PEDV-N protein was expressed as inclusion bodies then washed three times with PBS followed by reconstitution in 8 M urea (Sigma-Aldrich). All proteins were further purified using Ni+ affinity chromatography (Transgene, Beijing, China) and eluted with elution buffer then dialyzed as previously described (18). Dialysis of recombinant proteins was conducted using a gradient of decreasing urea concentration until the buffer was completely replaced by PBS or upon reaching a minimal urea concentration allowed without incurring visible protein precipitation during dialysis. The recombinant protein was quantified using a BCA protein assay kit (Thermo Fisher Scientific) and stored at -80°C until used for immunizations or to coat ELISA plates.

Mice were immunized three times at two-week intervals with 100 μg recombinant 6 × His-tagged PEDV-N protein (2 mg/mL) mixed with an equal volume of adjuvant. Freund’s complete adjuvant (Sigma-Aldrich) was used for the primary immunization, while Freund’s incomplete adjuvant (Sigma-Aldrich) was used for the remaining immunizations. Serum was collected from each mouse weekly for use in ELISA monitoring of antibody levels. Serum collected before immunization was included as a negative control.

For the generation of SLA-DR specific Mabs, the SLA-DRα Mab (Clone No.2E11D9) recognizing a liner epitope from SLA-DRα chain was previously described and verified (18). For the generation of Mab recognizing whole SLA-DR molecule, cell membrane protein was obtained from HEK 293T SLA-DRα^zsGreen^/β^mCherry^ stable cell line using Minute plasma membrane protein isolation kit (Invent Biotechnologies, Inc., Plymouth, MN, USA) according to the manufacturer’s instructions. Next, recombinant SLA-DRα immunized mice (18) were further immunized 100μg cell membrane extract for three additional times before cell fusion for hybridomas. After cell fusion, cell culture supernatant of the survived hybridomas was tested by IFA using PAMs (expressing SLA-DR) for positive clones, the reactivity of hybridomas to whole SLA-DR molecules were further verified using immune-precipitation assay to pull-down assembled SLA-DR (verified in western blot). Finally, the SLA-DR Mab (Clone No. MY5565) recognizing whole SLA-DR molecule was selected.

### Western Blot Analysis

Whole cell lysates of BM-DCs, Vero cells, HEK-293T cells, and cells of the HEK-293T-SLA-DR stable cell line were harvested using 1× Laemmli sample buffer (Bio-Rad Laboratories, Hercules, CA, USA). Next, lysate proteins were separated *via* sodium dodecyl sulfate-polyacrylamide gel electrophoresis (SDS-PAGE) as previously described (24). Separated proteins were then transferred to PVDF membranes as previously described (25). Membranes were probed with mouse serum raised against PEDV-N protein (1:200 dilution in TBS), homemade mAb against SLA-DRα (Clone No.2E11D9), homemade polyclonal antibodies against SLA-DRβ, anti-β-tubulin mAb (Transgene), or anti-ubiquitin mAb (Santa Cruz Biotech, Santa Cruz, CA, USA). Specific binding between antibodies and corresponding targets was detected based on binding to HRP-conjugated goat anti-mouse IgG (Thermo Fisher Scientific). Results were visualized using ECL substrate (Bio-Rad Laboratories), with chemiluminescent signal acquisition conducted using a ChemiDoc MP Imaging System (Bio-Rad Laboratories) and analysis of data conducted using Image Lab software (Version 5.1, Bio-Rad Laboratories).

### RNA Isolation and Quantitative Real-Time PCR

Total RNA was extracted from BM-DCs using TRIzol Reagent (Thermo Fisher Scientific) in accordance with the manufacturer’s instructions. Reverse transcription and qPCR were conducted using a PrimeScript RT reagent Kit (TaKaRa, Dalian, China) and 2× RealStar Power SYBR Mixture (Genstar, Beijing, China) respectively, as per the manufacturer’s instructions. Tubulin RNA transcript sequences were amplified from the same cDNA samples obtained from all groups to normalize total RNA input across samples. Primers used for qPCR and corresponding DNA sequences are listed in **Tables 1** and **2**. Relative quantification of target genes was calculated using the 2^−ΔΔCt^ method.

**Table 2:** List of primers using for qPCR.

Primers	Sequence (5’-3’)	Description
TubulinF	GGCTGTCTGCGATATCCCTC	qPCR for Tubulin
TubulinR	TGCTCTGAGATGCGCTTGA	
SLA-DRαF	GCCCTGAAGCCACTCTAA	qPCR for SLA-DRα
SLA-DRαR	GGAAAGCCAGCACAAGAA	
SLA-DRβF	GAGGGCACGGTCTGAATC	qPCR for SLA-DRβ
SLA-DRβR	AGGGCGTCCTTTCTGATT	
CD80-F	GGGAACACCATTACCCAAGC	qPCR for CD80
CD80-R	TCACCTGAACGATGCCTGA	qPCR for CD80
CADM1-F	GGCTTCTGCTGTTGCTCCTCT	qPCR for CADM1
CADM1-R	CGGAAGTCCCTGAAATAAATGGT	qPCR for CADM1
CD17-F	GCCCTGCGGACGTGGAGTTT	qPCR forCD17a
CD17a-R	GGGAGAAGCCGTGGGATTTGC	qPCR forCD17a
CD14-F	GCTCACCACCCTCAGACTCCGTAATGT	qPCR forCD14
CD14-R	GCGAGCTTGCTTGCGCCACTT	qPCR forCD14
DC-SIGN-F	TGCTCTTCGTCTCATTGGGTTTC	qPCR forDC-SIGN
DC-SIGN-R	TGTGGGTCTCCTGCTGGTCT	qPCR forDC-SIGN

### Immunoprecipitation and Protein Ubiquitination Assay

The immunoprecipitation-based protein ubiquitination assay for determining SLA-DR ubiquitination status was conducted as previously described with modifications (21). Briefly, 1 × 10^7^ BM-DCs infected with PEDV or mock-infected cells were harvested with 200 μL RIPA buffer (Thermo Fisher Scientific) supplemented with protease inhibitor cocktail (Sigma-Aldrich) and N-ethylmaleimide (NEM; Sigma-Aldrich) at a final concentration of 50 µM. The lysate was clarified by centrifugation at 14,000 ×*g* for five minutes at 4°C. Next, 3 μg of SLA-DR monoclonal antibody (Clone No. MY5565, recognizing the αβ heterodimer) was added to the cell lysate and the mixture was incubated at 4°C for three hours to permit mAb binding to SLA-DR. Next, 25 μL of protein G agarose beads (Genscript) was then added to the cell lysate followed by incubation for another hour to pull-down immune complexes. The IP supernatant was removed by centrifugation with 10,000 ×*g* at 4°C then the IP pellet was washed three times with RIPA buffer supplemented with protease inhibitor cocktail and NEM before harvesting the beads with 1 × Laemmli sample buffer for Western blot analysis. Western blots were probed with antibodies against ubiquitin to determine SLA-DR ubiquitination status in samples of all groups.

### Luciferase Reporter Assay

The promoter regions (−2000 nt to + 100 nt upstream of SLA-DR α and β genes) of SLA-DRα and β were cloned from genome DNA then ligated to pGL3.0 firefly luciferase reporter vectors and confirmed by DNA sequencing. Next, HEK293T cells were transfected with plasmids encoding PEDV proteins along with reporter vector pGL3.0-SLA-DRα or pGL3.0-SLA-DRβ. Renilla luciferase vector pRL-TK was also transfected for normalization. At 48 h after transfection, the cells were lysed using Cell lysis buffer (Promega) for luciferase activity assay of Firefly and Renilla luciferases using Dual-Glo^®^ Luciferase Assay System (Promega), by following manufacturer’s instructions. Lysate of cells transfected with empty vector of testing plasmids was used as a control for calculation of promoter’s activation level.

### Enzyme-Linked Immunosorbent Assay

For evaluation of mouse serum antibodies specific for PEDV-N protein, recombinant N proteins of different PEDV strains (400 ng/well) were used to coat wells of 96-well polystyrene microplates (Corning Inc.) in a volume of 100 μL PBS (pH 8.0) per well overnight at 4°C. Plates were blocked with 5% skim milk in PBS containing 0.5% Tween20 (Sigma-Aldrich). Diluted mice serum was added to wells then plates were incubated for one hour at 37°C followed by washing with PBS containing 0.5% Triton X-100 (Sigma-Aldrich). Binding of an antibody to its corresponding antigen was detected using HRP-conjugated goat anti-mouse IgG antibodies (GenScript) followed by visualization of results using a TMB substrate kit (Tiangen Biotech, Beijing, China). The absorbance of each well was measured using a Victor ™ X5 Multilabel Plate Reader (Perkin Elmer) at 450 nm.

### Fluorescence-Activated Cell Sorting

To analyze surface SLA-DR expression in BM-DCs after PEDV infection, 1 × 10^6^ BM-DCs were inoculated with PEDV, incubated for 24 h, then cells were stained with anti-SLA-DR Mab (Clone No. MY5565, recognizing the αβ heterodimer) as the primary antibody followed by addition of APC-conjugated goat anti-mouse cross-adsorbed secondary antibody (Thermo Fisher Scientific). Flow cytometric analyses were performed using a FACS Aria™ III cell sorter (BD Biosciences) using FlowJo software, version 10.0.7 (Tree Star, Ashland, Oregon, USA).

### Immunofluorescence Assay

PEDV-infected or non-infected Vero cells and BM-DCs in cell culture plates were fixed with 4% paraformaldehyde (Sigma-Aldrich), permeabilized with PBS containing 0.5% Triton X-100 (Sigma-Aldrich), then blocked with PBS containing 1% BSA (Sigma-Aldrich). Next, the cells were stained with mouse serum against PEDV-N protein. Specific binding between antibodies and corresponding targets was detected using Alexa Fluor^®^ 555-labeled goat anti-mouse IgG (Thermo Fisher Scientific). Cellular nuclei were counterstained with 4’,6-diamidino-2-phenylindole (DAPI; Thermo Fisher Scientific) at 37°C for 10 min and observed under a Leica DM1000 fluorescence microscope (Leica Microsystems, Wetzlar, Germany). All images were captured and processed using Leica Application Suite X (Leica Microsystems).

### Statistical Analysis

Results were analyzed using GraphPad Prism version 5.0 (GraphPad Software, San Diego, CA, USA). Statistical significance was determined using either Student’s *t*-test for the comparison of two groups or one-way analysis of variance (ANOVA) for testing of more than two groups. A two-tailed *P* value < 0.05 was considered as statistically significant.

## Results

### Bone Marrow-Derived Dendritic Cells Were Susceptible to PEDV Infection

**Figure 1:**
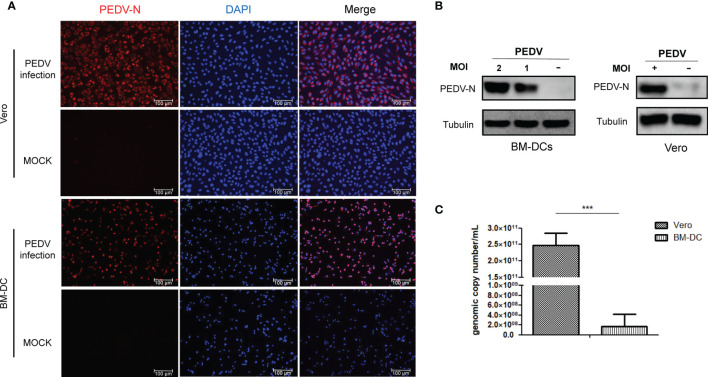
BM-DCs is susceptible for PEDV and supports PEDV replication *in vitro*. **(A)** Vero cells and BM-DCs were infected by Vero cell-adapted PEDV strain KB2013-p120 at 1MOI for 24 hours then harvested and subjected with immunofluorescence assay using mice serum raised against recombinant PEDV-N protein and visualized by secondary antibodies. Representing IFA images were selected from at least three independent experiments (hereby and thereafter unless specified). **(B)** BM-DCs were infected by PEDV-KB2013-p120 strain at 1MOI or 2MOI for 24 hours then harvested for western blot using mice serum raised against recombinant PEDV-N protein. Normal BM-DCs cells without PEDV infection were included as control. The Vero cells infected by PEDV-KB2013-p120 strain at 1MOI for 24 hours then harvested for western blot using mice serum raised against recombinant PEDV-N protein. Normal Vero cells without PEDV infection were included as control. Representing western blot images were selected from at least three independent experiments (hereby and thereafter unless specified). **(C)** BM-DCs and Vero cells were infected by PEDV-KB2013-p120 strain at 1MOI for 24 hours then harvested by TRizol for absolute quantification of PEDV viral RNA copies using qPCR. Error bars represent variation from at least three independent experiments. Significant differences between indicated groups was marked by ***P < 0.001.

Antagonism of the host innate immune response by PEDV has been extensively investigated (26), although few studies have focused on underlying mechanisms responsible for PEDV antagonism of host adaptive immune response. A major obstacle has been the lack of *in vivo* isolated dendritical cells for use in conducting *in vitro* PEDV infection experiments, due to difficulties associated with dendritical cells isolation. In addition, conflicting results have been obtained from previous *in vitro* virus infection studies using *in vitro* differentiated DCs such as monocyte-derived dendritic cells (MoDCs) (13, 14). Nevertheless, in our previous study we successfully developed monoclonal antibodies against SLA-DR and used BM-DCs to elucidate underlying mechanisms involved in porcine reproductive and respiratory syndrome virus (PRRSV) promotion of SLA-DR expression (18, 27), prompting the application of BM-DC cells here as an APC model of PEDV infection. To determine if BM-DCs were susceptible to PEDV infection, a Vero cell-adapted PEDV strain was added to BM-DCs (MOI=1) then KB2013-p120 was added to co-cultures of BM-DCs and Vero cells followed by incubation for 24 h. Next, sera of BALB/C mice (collected after a third immunization with recombinant PEDV-N protein, **Figure S1**) was used as a probe to detect virus within cells using an immunofluorescence assay (IFA) to determine virus numbers. As demonstrated in **Figure 1A**, immunostained Vero cells exhibited a strong positive signal for PEDV-N antigen and most BM-DCs were positive for PEDV-N antigen as well, although signals associated with BM-DCs were weaker than signals associated with Vero cells (**Figure 1A**). Thus, BM-DCs were also susceptible to PEDV infection, but were less susceptible than Vero cells. In order to confirm this result, Western blot analysis was conducted to detect PEDV-N protein as well. Similarly, PEDV-N protein in infected BM-DCs was detectable *via* Western blotting and levels increased with increasing MOI of PEDV used to inoculate BM-DCs (**Figure 1B**). Meanwhile, viral RNA copies in PEDV infected BM-DCs and Vero cells were evaluated by absolute quantification using qPCR. As demonstrated in **Figure 1C**, viral RNA level in Vero cells was 1000 folds more than that of in BM-DCs, further suggested that BM-DCs less susceptible to PEDV than Vero cells. Taken together, the abovementioned results suggested that BM-DCs generated *in vitro* were susceptible to PEDV infection.

### PEDV Infection of BM-DCs Inhibited Expression of SLA-DR

**Figure 2:**
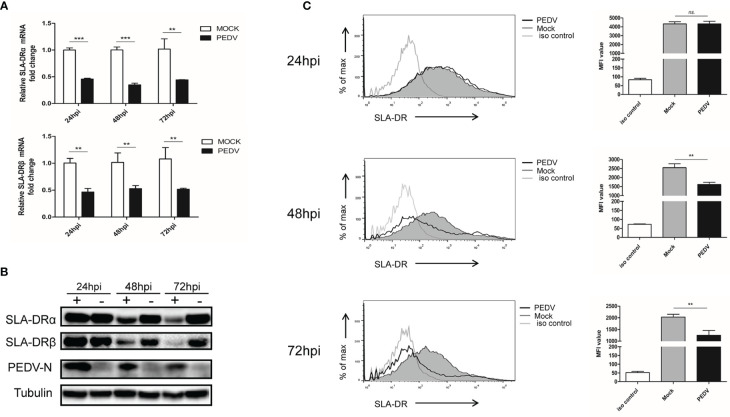
PEDV replication inhibit expression of SLA-DR in BM-DCs **(A)** BM-DCs were infected by Vero cell-adapted PEDV strain KB2013-p120 at 1MOI for 24, 48 and 72 hours then harvested for qPCR analysis for mRNA level of SLA-DRα and SLA-DRβ. Transcript of tubulin were analyzed from the same sample to normalize total RNA input. Error bars represent variation from at least three independent experiments. Significant differences between indicated groups was marked by **P < 0.01; ***P < 0.001. **(B)** BM-DCs were infected by PEDV-KB2013-p120 strain at 1MOI for 24, 48 and 72 hours then harvested for western blot to evaluate SLA-DRα, SLA-DRβ and PEDV-N protein level using corresponding antibodies. Normal BM-DCs cells without PEDV infection were included as control. Tubulin was probed from the same sample to normalize the total protein load. **(C)** BM-DCs were infected by PEDV-KB2013-p120 strain 1MOI for 24, 48 and 72 hours then stained with anti-SLA-DR antibody followed by visualization of APC labeled goat anti-mouse IgG. Then the cells were subjected to flow cytometry analysis for evaluating cell surface expression of SLA-DR. BM-DCs without PEDV infection stained with normal mouse IgG as primary antibody were included as primary antibody isotype control. Error bars represent variation of quantification of FACS data from at least three independent experiments. Significant differences between indicated groups was marked by **P < 0.01; or ns, non significant.

A typical characteristic of APCs is their expression of MHC-II molecules that present antigens to CD4+ T helper cells to trigger adaptive immune responses (15, 16). In swine species, swine leukocyte antigen-DR (SLA-DR) serves as major player responsible for foreign-antigen presentation (27, 28). Therefore, SLA-DR expression levels were first investigated here. Based on our results, after BM-DCs infection with PEDV, SLA-DRα and SLA-DRβ mRNA levels each decreased significantly, with reductions exceeding 50% from 24 hours post-inoculation (hpi) to 72 hpi (**Figure 2A**). In alignment with reduced mRNA levels, SLA-DRα and SLA-DRβ protein levels were significantly reduced in PEDV-infected BM-DCs as compared to corresponding levels in uninfected BM-DCs (**Figure 2B**).

Importantly, after assembly of SLA-DR heterodimers from α and β chains, heterodimers must be transported from the cytoplasm to the cell surface of APCs to carry out their biological function of presenting antigenic peptides within the SLA-DR peptide cleft to CD4+ T helper cells. Therefore, flow cytometry was conducted using a homemade Mab that recognized assembled SLA-DR heterodimers (but not unassembled SLA-DRα or β chains) in order to assess surface expression of assembled SLA-DR on BM-DCs. As demonstrated in **Figure 2C**, markedly reduced levels of assembled SLA-DR were observed on surfaces of PEDV-infected BM-DCs as infection times increased from 24 hpi to 72 hpi, implying that antigen presentation capability of PEDV-infected BM-DCs was strongly impaired. Therefore, these results suggested that PEDV infection of BM-DCs strongly inhibited SLA-DR expression and reduced levels of membrane surface SLA-DR molecules, potentially preventing infected BM-DCs from functioning as APCs.

### Bone Marrow-Derived Dendritic Cells and *In Vivo* Isolated Macrophages Were Permissive for PEDV Infection

**Figure 3:**
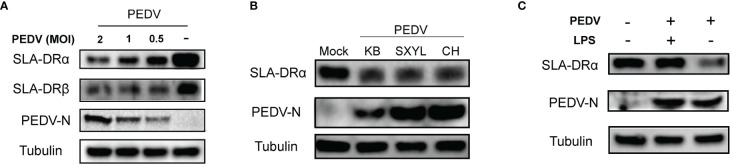
Susceptibility of BM-DCs for PEDV is not strain-specific. **(A)** BM-DCs were infected by PEDV-KB2013-p120 strain at 0.5, 1 and 2MOI for 48 hours then harvested for western blot to evaluate SLA-DRα, SLA-DRβ and PEDV-N protein. Normal BM-DCs cells without PEDV infection were included as control. Tubulin was probed from the same sample to normalize the total protein load. **(B)** BM-DCs were infected by Vero cell-adapted PEDV strain KB2013-p120, virulent PEDV strain CH/hubei/2016 (GenBank accession number: KY496315.1), and a field PEDV isolate SXYL-21 for 48 hours. Next, cells were harvested for western blot using mice serum raised against recombinant PEDV-N protein and anti-SLA-DRα Mab. Normal BM-DCs cells without PEDV infection were included as control. Tubulin was probed from the same sample to normalize the total protein load. **(C)** BM-DCs were infected by PEDV-KB2013-p120 strain at 1 MOI for 24 hours then 10μg LPS was added to the PEDV infected BM-DCs to stimulate the cells for another 24 hours. Next, all cells were harvested for western blot to evaluate SLA-DRα, SLA-DRβ and PEDV-N protein. PEDV infected BM-DCs without LPS stimulation and normal BM-DCs cells were included as controls. Tubulin was probed from the same sample to normalize the total protein load.

As indicated by the abovementioned results, PEDV-KB2013-p120, a Vero cell-adapted PEDV strain, could infect BM-DCs and inhibit SLA-DR expression when infecting BM-DCs since dose-dependent PEDV inhibition of SLA-DRα and SLA-DRβ protein levels was observed, with inhibition increasing with increased PEDV MOI used to inoculate BM-DCs (**Figure 3A**). Thus, we investigated whether susceptibility of BM-DCs to PEDV is a universal phenomenon or is virus strain-specific, prompting us to inoculate BM-DCs with other PEDV strains in addition to KB2013-p120. Additional PEDV strains included the virulent PEDV strain CH/hubei/2016 strain isolated during a PED outbreak in Hubei province in 2016 and the currently circulating PEDV strain SXYL-21 (full sequence unavailable) isolated from a nearby pig farm this year. As demonstrated in **Figure 3B**, all PEDV-infected BM-DCs harbored detectable PEDV-N antigen regardless of PEDV strain used for inoculation. It was also notable that PEDV-N protein levels in PEDV-KB2013-p120-infected BM-DCs were relatively lower than levels in CH/hubei/2016- and SXYL-21-infected BM-DCs (**Figure 3B**), implying that replication levels of a Vero cell-adapted PEDV strain (attenuated for swine, data not shown) in BM-DCs were lower than replication levels of virulent PEDV strains. Nevertheless, levels of inhibition of SLA-DRα expression by various PEDV isolates appeared to be similar (**Figure 3B**). With regard to SLA-DRβ expression, BM-DCs used in this study were *in vitro* differentiated cells derived from primary bone marrow cells collected from multiple pigs with multiple SLA-DRB alleles with greater diversity than SLA-DRA alleles of pigs used to generate BM-DCs (29). Thus, additional SLA-DRβ variants encoded by diverse SLA-DRB alleles might not have been detectable using our mouse serum raised against recombinant SLA-DRβ, as evidenced by our observation that the serum did not react with SLA-DRβ of swine used to generate PAMs and bone marrow-derived cells for this analysis (data not shown). Therefore, protein expression levels of SLA-DRβ in BM-DCs could not be confirmed in this work. Meanwhile, it was also notable that PEDV mediated inhibition of SLA-DR could be rescue by LPS treatment of DCs (**Figure 3C**).

**Figure 4:**
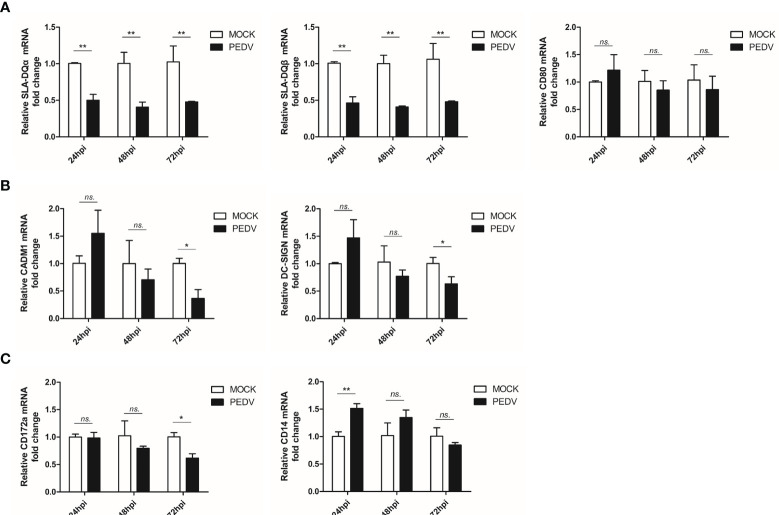
PEDV infection of BM-DCs alternated expression of antigen presentation related molecules **(A)** BM-DCs were infected by PEDV-KB2013-p120 strain 1 MOI for 24, 48 and 72 hours, then cells were harvested by TRizol for qPCR analysis of SLA-DQα, SLA-DQβ and CD80. Normal BM-DCs cells without infection harvested at the same time-points were included as control. **(B)** BM-DCs were infected by PEDV-KB2013-p120 strain 1 MOI for 24, 48 and 72 hours, then cells were harvested by TRizol for qPCR analysis of CDAM1 and DC-SIGN. Normal BM-DCs cells without infection harvested at the same time-points were included as control. **(C)** BM-DCs were infected by PEDV-KB2013-p120 strain 1 MOI for 24, 48 and 72 hours, then cells were harvested by TRizol for qPCR analysis of CD172a and CD14. Normal BM-DCs cells without infection harvested at the same time-points were included as control. Error bars represent variation from at least three independent experiments. Significant differences between indicated groups was marked by *P < 0.05; **P < 0.01. or ns, non significant.

Conversely, to further investigate if PEDV replication in BM-DCs impairs their APC function, mRNA level of other antigen presentation related molecules, such as SLA-DQ, CD80, CADM1, DC-SIGN, CD172a and CD14 were examined. As demonstrated in **Figure 4A**, SLA-DQα and β expression were still inhibited by PEDV, which was similar to that of SLA-DR, whereas expression of co-stimulation molecule CD80 was unchanged (**Figure 4A**). Moreover, for other DCs-specific molecules involved in DC-T cell interaction, such as CADM1 and DC-SIGN, no significant changes of CADM1 and DC-SIGN mRNAs could be observed in 24 and 48 hours after PEDV infection in BM-DCs (**Figure 4B**), whereas a slight inhibition of CADM1 and DC-SIGN mRNAs could be observed after 72 hours after PEDV infection (**Figure 4B**). For other surface markers of DCs, such as CD172a and CD14, an inhibition of CD172 mRNA was observed 72 hours after PEDV infection (**Figure 4C**), whereas an evaluation of CD14 could be observed in 24 hours after PEDV infection (**Figure 4C**).

**Figure 5:**
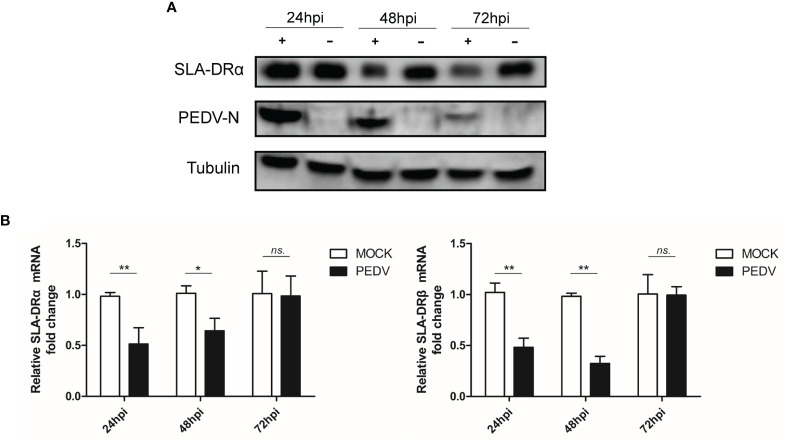
PAMs are susceptible for PEDV. **(A)** PAMs were infected by Vero cell-adapted PEDV strain KB2013-p120 for 24, 48 and 72 hours. Next, cells were harvested for western blot using mice serum raised against recombinant PEDV-N protein and anti-SLA-DRα Mab. Normal PAMs cells without PEDV infection were included as control. Tubulin was probed from the same sample to normalize the total protein load. **(B)** PAMs were infected by PEDV strain KB2013-p120 for 24, 48 and 72 hours. Next, cells were harvested by TRizol for qPCR analysis of SLA-DRα and β mRNA level. Normal PAMs cells without PEDV infection were included as control. Error bars represent variation from at least three independent experiments. Significant differences between indicated groups was marked by *P < 0.05; **P < 0.01. or ns, non significant.

Since BM-DCs used here as APCs were generated *in vitro*, we tested primary porcine alveolar macrophages (PAMs) collected from pig lungs for susceptibility to infection with PEDV-KB2013-p120. Our results suggested that *in vivo*-isolated PAMs were permissive for PEDV infection (**Figure 5A**) and that SLA-DRα expression was inhibited in these cells as well (**Figure 5B**), mirroring BM-DCs results obtained here. Taken together, these results suggested that both *in vitro*-cultured BM-DCs and *in vivo*-isolated PAMs were susceptible to PEDV infection regardless of PEDV strain.

### PEDV Replication Is Not Required for Inhibition of SLA-DR Expression in BM-DCs

**Figure 6:**
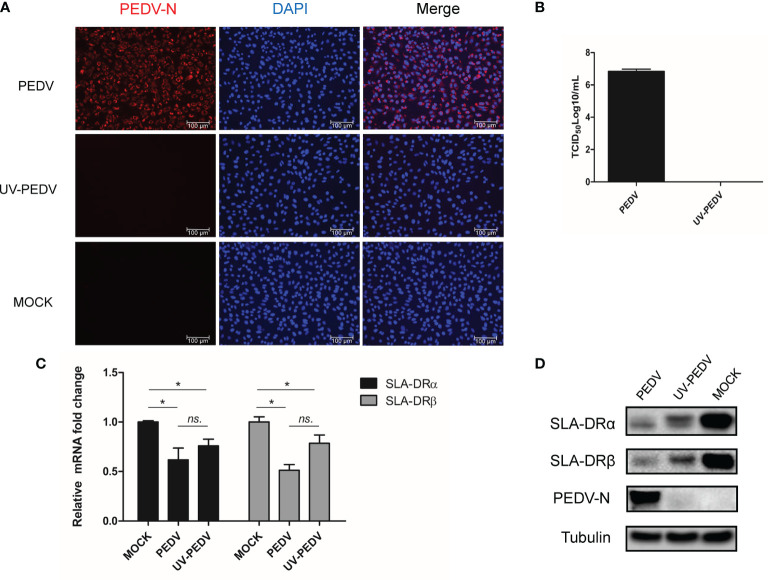
Inhibition of SLA-DR expression in BM-DCs did not require PEDV replication. **(A)** BM-DCs were inoculated with either live PEDV-KB2013-p120 strain or UV-inactivated PEDV-KB2013-p120 strain for 24 hours. Next the cells were fixed and stained with mice serum raised against recombinant PEDV-N protein and visualized by secondary antibody. **(B)** BM-DCs were inoculated with either live PEDV-KB2013-p120 strain (PEDV) or UV-inactivated PEDV-KB2013-p120 strain (UV-PEDV) for 24 hours. Next, cell culture supernatants were harvested and titrated in Vero cells. Error bars represent variation from at least three independent experiments. **(C)** BM-DCs were inoculated with either live PEDV-KB2013-p120 strain (PEDV) or UV-inactivated PEDV-KB2013-p120 strain (UV-PEDV) for 24 hours. Next, cells were harvested for qPCR evaluation of mRNA level of SLA-DR α and SLA-DR β. All experiment was repeated at least for three times. Significant differences between indicated groups was marked by *P < 0.05. **(D)** BM-DCs were inoculated with either live PEDV-KB2013-p120 strain (PEDV) or UV-inactivated PEDV-KB2013-p120 strain (UV-PEDV) for 48 hours. Next, cells were harvested for western blot using mice serum raised against recombinant PEDV-N protein, anti-SLA-DRα Mab and mice serum raised against recombinant SLA-DRβ protein. Normal BM-DCs cells were included as control. Tubulin was probed from the same sample to normalize the total protein load. ns, non significant.

Although the abovementioned data suggest that PEDV could replicate in BM-DCs and cause down-regulation of SLA-DR, we noticed that PEDV replication in BM-DCs did not induce visible cytopathic effect (CPE), as severe CPE could be observed in PEDV infected Vero cells (**Figure S2**). These results suggested that levels of PEDV replication in BM-DCs were lower than levels of PRRSV replication in BM-DCs, which was further supported by qPCR quantification of PEDV RNA copies in PEDV infected Vero and BM-DCs (**Figure 1C**). Therefore, it would be interesting to investigate whether PEDV-mediated SLA-DR inhibition required active PEDV replication, prompting us to inoculate BM-DCs with live PEDV or UV-inactivated PEDV in parallel assays. Ultimately, UV-inactivated PEDV (confirmed in Vero cells, **Figure S2**) was unable to replicate in BM-DCs, as shown by a lack of detectable PEDV-N protein in BM-DCs inoculated with UV-inactivated PEDV (**Figure 6A**). Moreover, no viral replication (as determined by TCID_50_) could be detected in Vero cells if further inoculated Vero cells using cell culture supernatant obtained from BM-DCs inoculated by UV-inactivated PEDV (**Figure 6B**), suggested that PEDV was complete inactivated by UV. Nevertheless, qPCR analysis suggested that BM-DCs inoculated with either live PEDV or UV-inactivated PEDV demonstrated significant reductions of SLA-DRα and SLA-DRβ mRNA levels, with BM-DCs infected with live PEDV exhibiting enhanced reduction of both SLA-DRα and SLA-DRβ mRNAs (**Figure 6C**). In alignment with these results, SLA-DRα protein levels were reduced in BM-DCs inoculated with either live PEDV or UV-inactivated PEDV, with greater inhibition of SLA-DRα and β protein expression observed in live PEDV-infected BM-DCs as well (**Figure 6D**). Taken together, these data suggested that UV-inactivated PEDV inhibited BM-DCs expression of SLA-DR, implying that PEDV structural proteins within viral particles may be responsible for SLA-DR expression inhibition rather than non-structural virus proteins.

### PEDV Envelope Protein Inhibited Activation of SLA-DRα and β Promoters

**Figure 7:**
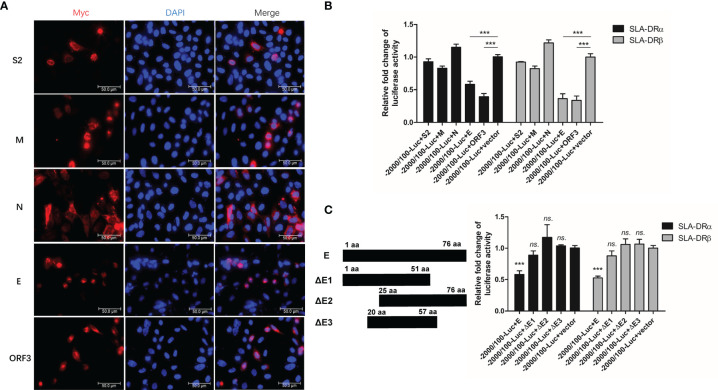
PEDV-Envelope and ORF3 proteins inhibit promoters’ activation of SLA-DR. **(A)** HEK-293T cells were transfected with plasmids encoding S2, M, N, E and ORF3 for 48 hours. Next, cells were fixed and stained with anti-C-MYC tag Mab and visualized by secondary antibody. **(B)** HEK-293T cells were transfected with plasmids encoding S2, M, N, E and ORF3, along with firefly luciferase reporter plasmids bearing promoters of SLA-DRα and SLA-DRβ for 48 hours. Control plasmid pRL-TK was co-transfected to normalize transfection. Next, cells were harvested for evaluation of luciferase activity. HEK-293T cells transfected with empty vector and luciferase reporters were included as controls. Error bars represent variation from at least three independent experiments. Significant differences of luciferase activity between indicated groups was marked by ***, *P* < 0.001. **(C)** Schematic illustration of truncations of PEDV-E protein. HEK-293T cells were transfected with plasmids encoding full length PEDV-E protein and truncations, along with firefly luciferase reporter plasmids bearing promoters of SLA-DRα and SLA-DRβ for 48 hours. Control plasmid pRL-TK was co-transfected to normalize transfection. Next, cells were harvested for evaluation of luciferase activity. HEK-293T cells transfected with empty vector and luciferase reporters were included as controls. Error bars represent variation from at least three independent experiments. Significant differences of luciferase activity between cell groups transfected with E truncations and empty vector was marked by ***P < 0.001, or “ns.” means nonsignificant.

Since the abovementioned data suggested that PEDV structural proteins could be involved in downregulation of SLA-DR expression, a reporter-based assay was used to monitor promoter activities. All PEDV genes except nsps, including the gene encoding accessory protein ORF3 and genes encoding structural proteins, were cloned into mammalian expression vectors and fused to a C-MYC tag when expressed in HEK-293T cells. Except for the S1 fragment of PEDV-S protein, expression of all other proteins was confirmed using IFA (**Figure 7A**) and Western blotting (**Figure S3**). Next, plasmids containing genes encoding these PEDV proteins were co-transfected into HEK293T cells with firefly luciferase reporter-based constructs containing promoters (−2000 nt to + 100 nt upstream of SLA-DR α and β genes) along with transfection control plasmid pRL-TK. Analysis of luciferase activity suggested that PEDV-ORF3 and envelope (E) protein were the strongest inhibitors of promoter activities of genes encoding both SLA-DR α and β (**Figure 7B**). E protein is a known structural protein present within PEDV virions. To more precisely localize the E protein region involved in inhibition of promoter activity, different truncations of PEDV-E protein were generated (**Figure 7C**) and screened in reporter assays as well. However, no truncations of PEDV-E protein could inhibit SLA-DRα and β promoter activities, suggesting that full-length PEDV-E protein may be required for transcriptional inhibition of SLA-DR genes (**Figure 7C**). Taken together, these results indicated that PEDV-E protein and ORF3 protein were responsible for silencing promotors controlling SLA-DRα and β gene transcription, thus regulating gene expression at transcriptional level. Additionally, full-length PEDV-E protein played a major role in inhibiting activation of SLA-DRα/β promoters after inoculation of BM-DCs with UV-inactivated PEDV, while ORF3 presented during PEDV replication to further silence SLA-DRα/β promoter activities.

### Inhibition of SLA-DR Expression Did Not Involve Ubiquitin-Proteasome Pathway-Mediated Degradation of SLA-DR at the Protein Level

**Figure 8:**
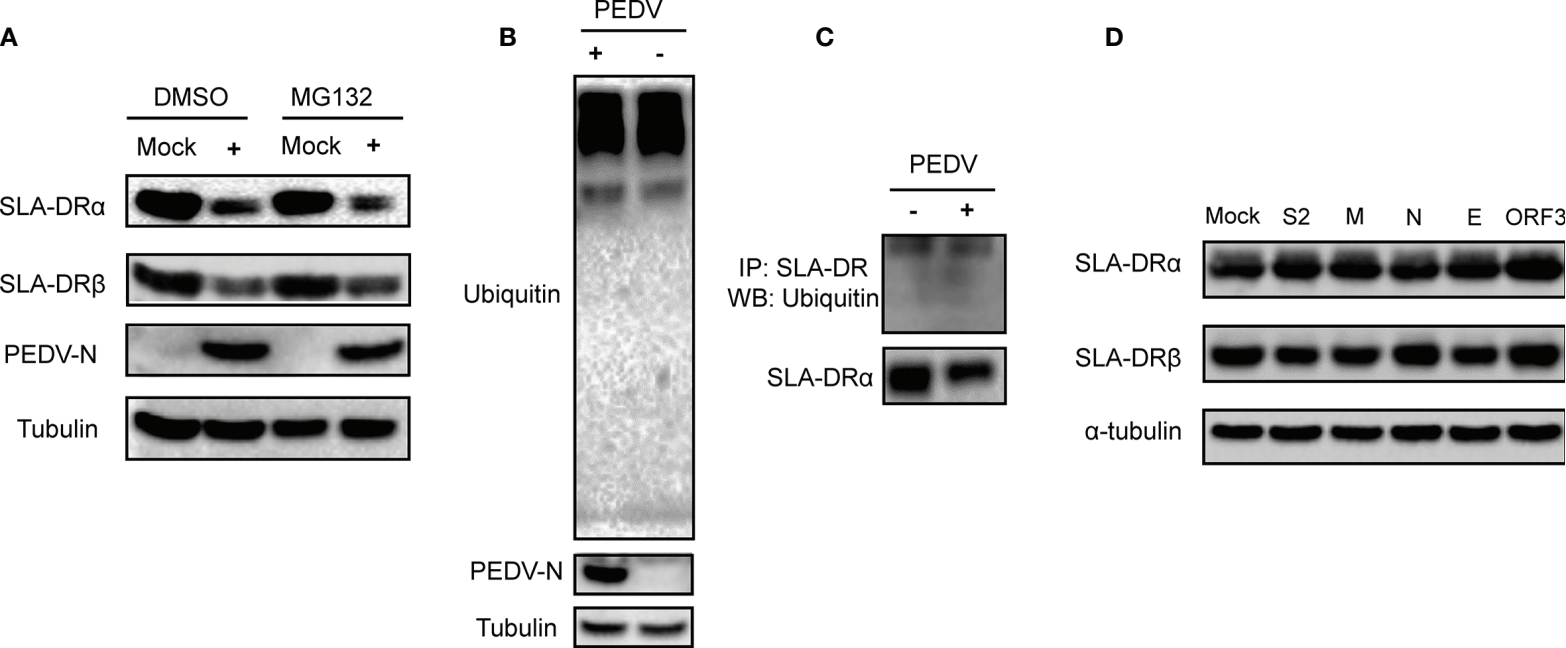
Ubiquitin-proteasome pathway did not involve in inhibition of SLA-DR expression. **(A)** BM-DCs were infected by PEDV-KB2013-p120 strain at 1MOI for 24, then followed by treatment of either DMSO or MG132 for another 24 hours. Then cells were harvested for western blot evaluating of SLA-DRα, SLA-DRβ and PEDV-N protein. Normal BM-DCs cells without PEDV infection but treated with the same dose of DMSO or MG132 were included as controls. Tubulin was probed from the same sample to normalize the total protein load. **(B)** BM-DCs were infected by PEDV-KB2013-p120 strain at 1MOI for 24 hours. Next, cells were harvested for western blot evaluating of universal ubiquitination level and PEDV-N protein. Normal BM-DCs cells were included as control. Tubulin was probed from the same sample to normalize the total protein load. **(C)** BM-DCs were infected by PEDV-KB2013-p120 strain at 1MOI for 24 hours. Next, cells were lyzed using RIPA buffer supplemented with protease inhibitor cocktail and NEM for SLA-DR enrichment using anti-SLA-DR Mab-MY533. Enriched samples were subjected to western blot using ubiquitin antibody and anti-SLA-DRα chain antibody. **(D)** HEK-293T-SLA-DRα/β stable cells were transfected with plasmids encoding S2, M, N, E and ORF3 for 48 hours. Next, cells were harvested for western blot to evaluate expression of SLA-DRα and β. Cells transfected with empty vector were included as control. Tubulin was probed from the same sample to normalize the total protein load.

Since the abovementioned data suggested that PEDV envelope protein could inhibit transcription of SLA-DRα/β-encoding genes in the absence of viral replication, we investigated whether inhibition of SLA-DR expression in PEDV-infected cells occurred at the protein level as well. Therefore, the ubiquitin-proteasome pathway, a protein degradation pathway targeted by many viruses to control protein half-life (30), was investigated for its potential role in PEDV-mediated SLA-DR inhibition. After treatment of PEDV-infected BM-DCs with MG132, a proteasome inhibitor, MG132 inhibition of the ubiquitin-proteasome pathway did not rescue either SLA-DRα or SLA-DRβ protein-level expression, implying that reduced SLA-DR protein levels in PEDV-infected BM-DCs were solely due to transcriptional inhibition (**Figure 8A**). To confirm this speculation, investigation of total ubiquitination status of PEDV-infected BM-DCs was conducted, with results obtained that confirmed that the overall ubiquitination level of proteins in BM-DCs was not affected by PEDV infection (**Figure 8B**). Moreover, when SLA-DR molecules enriched from PEDV-infected BM-DCs using corresponding antibodies were probed with ubiquitin-specific antibody, SLA-DR ubiquitination levels in BM-DCs were similar regardless of PEDV infection status (**Figure 8C**). Furthermore, results of additional experiments using the HEV-293T-SLA-DR stable cell line generated by lentivirus transduction of SLA- DRα/β chain genes indicated that expressed SLA-DRα/β chains properly assembled to form functional SLA-DR heterodimers (**Figures S4A, B**). After transfecting PEDV structural proteins and ORF3 protein into cells of the HEV-293T-SLA-DR stable cell line, no alteration of SLA-DRα/β level was observed (**Figure 8D**), suggesting that these virus proteins did not alter SLA-DR expression at the protein level. Taken together, the aforementioned evidence indicates that reduced SLA-DR expression in BM-DCs inoculated with UV-inactivated PEDV was solely a consequence of transcriptional inhibition of SLA-DR, ruling out involvement of the ubiquitin-proteasome pathway in reduced SLA-DR expression associated with PEDV infection. Ultimately, reductions of cell surface SLA-DR heterodimers resulted from inhibition of transcription-level expression of both SLA-DRα and β chain genes due to strong inhibition of corresponding promoter activities by PEDV-E protein and additional inhibition (to a lesser degree) by PEDV-ORF3 protein during PEDV replication.

## Discussion

Porcine epidemic diarrhea virus (PEDV) has had catastrophic impacts on the global pig industry. Although the fecal–oral route is widely accepted as the major pathway involved in PEDV transmission between susceptible hosts, increasing evidence suggests that airborne transmission may contribute to PEDV outbreaks as well (31–33). In fact, in a recent report it was suggested that PEDV could cause typical diarrhea in piglets when administered *via* a nasal spray. This result indicated that PEDV may develop as a transient nasal epithelium infection that subsequently infects dendritic cells (DCs), which transfer the virus to CD3+ T cells *via* virological synapses (31). Eventually, virus-loaded CD3+ T cells reach the intestine through the blood circulation, leading to intestinal infection *via* cell-cell contact (31).

To date, *in vivo* PEDV infection of APCs such as DCs has not yet been confirmed, but may occur within Peyer’s patches, the major organized lymphoid structures involved in the induction of mucosal immune responses within the intestine. Peyer’s patches are comprised of dome-shaped arrangements of transmucosal clusters of lymphoid follicles and dendritic cells (DCs) such that antigen-presenting cells (APCs) reside predominantly within the dome and interfollicular Peyer’s patch areas (34). Notably, authors of one research report have detected PEDV antigens within APCs such as macrophages residing within the lamina propria and within Peyer’s patches in an *in vivo* PEDV infection study (2). However, *in vitro* studies using monocyte-derived dendritic cells (MoDCs) have produced conflicting results (13, 14). While both reports confirmed that inoculation of MoDCs by PEDV evoking secretion of cytokines (13, 14), such as IFN-γ and type I IFN, the later report failed to detected the viral antigen from PEDV inoculated MoDCs and qPCR analysis suggested that viral RNA copies decreased though time of infection (14). In our study, it was notable that PEDV-N antigen in PEDV infected BM-DCs and PAMs decreased though the infecting time. Although the reason for such observation was unclear, it is partially consisted with the previous observation that viral RNA decreased in PEDV-infected MoDCs as infecting time increased. However, our available data presented in here in favor of the fact that APCs are susceptible for PEDV.

Nevertheless, it is notable that the latest study demonstrated that dendritic cell-specific ICAM-grabbing non-integrin (DC-SIGN), a mannose-specific C-type lectin mainly expressed in immature DCs within the dermis, lymph nodes, and tonsils (35), renders cell lines (e.g., BHK-21) susceptible to PEDV entry and infection; conversely, blockage of DC-SIGN with mannan inhibited PEDV infection of cells bearing porcine DC-SIGN (36) as evidence that DC-SIGN is a potential receptor or factor involved in PEDV infection. Meanwhile, as for other well-characterized receptors, DC-SIGN has been shown to be involved in infections caused by other types of viruses, including coronavirus SARS-CoV2 (37, 38). As a result, these data imply that DCs are potential target cells of PEDV infection *in vivo*. Aligning with this speculation, our results here suggested that porcine BM-DCs were susceptible to infection by various PEDV strains as reflected by detectable PEDV-N protein levels in infected BM-DCs. However, cell susceptibility to virus infection varied depending on which PEDV strain was used to inoculate the cells, since it is possible that different PEDV strains possess variable tropisms for BM-DCs due to unknown factors, pending further investigation. Moreover, our results here demonstrated that primary cultures of porcine alveolar macrophages (PAMs) directly isolated from lung lavage specimens without further *in vitro* manipulations were also susceptible to PEDV infection. Therefore, collectively these data suggest that APCs are susceptible to PEDV infection and could act as *in vivo* targets of PEDV infection.

PEDV-induced inhibition of innate immune responses, such as type I IFNs induction and other signaling pathways, have been investigated for many years. It has been reported that PEDV encodes several proteins that act as IFNs antagonists as follows: nsp1, which degrades CREB-binding protein to suppress IFNs production (39); nsp15 endoribonuclease, which degrades mRNA of TBK1 and IRF3 (40); ORF3, which regulates IκB kinase β-mediated NF-κB and IFN-β promoter activation (41); structural protein N, which sequesters the interaction between IRF3 and TBK1 to block IFNs induction (42); and M protein, which interacts with IRF7 to inhibit IFNs production (43). Notably, an accumulating body of evidence suggests that cells involved in the adaptive immune response, such as DCs, are susceptible to PEDV infection as well. However, mechanisms by which PEDV interferes with the host adaptive immune response have not yet been thoroughly investigated.

Induction of pathogen-specific antibody responses requires activation of antigen-specific B cells with assistance of activated CD4+ T helper cells (15, 16). CD4+ T helper cells are activated by TCR-based recognition of MHC-II-mediated antigens presented by APCs (e.g., DCs). Generally, exogenous antigens are internalized by DCs *via* several pathways, including phagocytosis, macropinocytosis, and endocytosis, with antigens eventually trafficking to mature or late endosomal compartments where they are processed and loaded onto MHC-II molecules (44, 45). Meanwhile, DCs can also engage in autophagy-associated engulfing of cytosolic macromolecules and organelles that leads to autophagosomes formation followed by autophagosomes participation in membrane fusion with lysosomal compartments to generate peptide–MHC class II complexes (44, 45). In swine, SLA-DR, the counterpart of human leukocyte antigen-DR (HLA-DR), serves as a major player responsible for foreign antigen presentation and cross-talk with CD4+ T helper cells that are required to trigger adaptive immune responses (27, 28). Therefore, here we specifically focused on evaluation of SLA-DR expression status in BM-DCs after PEDV infection. Unexpectedly, PEDV-infected BM-DCs exhibited strong inhibition of mRNA-level and protein-level SLA-DR expression as compared to uninfected controls. Meanwhile, treatment of PEDV-infected BM-DCs with MG132, a ubiquitin-proteasome degradation pathway inhibitor, was unable to rescue protein-level expression of SLA-DR, suggesting that reduced SLA-DR levels were not due to accelerated degradation of SLA-DR *via* the ubiquitin-proteasome pathway. Moreover, it was also notable that UV-inactivated PEDV induction of SLA-DR degradation mirrored that induced by live PEDV, implying that PEDV structural proteins might participate in inhibition of SLA-DR expression in BM-DCs. However, our results here differed from previously reported findings showing that treatment of cells (MoDCs) with UV-inactivated PEDV did not affect SLA-DR expression (46), with conflicting results possibly due to physiological differences between BM-DCs and MoDCs.

After detailed analysis, our data suggested that inhibition of SLA-DR expression in BM-DCs by inactivated PEDV was potentially due to effects of PEDV envelope (E) protein. Support for this speculation was provided by results showing inhibition of SLA-DRα and SLA-DRβ promoter activities when PDEV-E protein overexpression was induced in HEK-293T cells, as confirmed using luciferase reporter plasmids containing SLA-DRα and SLA-DRβ promoters. PEDV-E protein is the smallest PEDV structural protein, with a molecular weight of about 7 kDa and relatively low-level homology to other coronavirus proteins. Although the mechanism by which PEDV-E protein inhibited SLA-DRα and SLA-DRβ promoter activation is unclear, one report has suggested that PEDV-E protein may induce endoplasmic reticulum (ER) stress and activate the nuclear factor-κB (NF-κB) pathway *in vitro* (47) to potentially regulate SLA-DR expression. Meanwhile, results of another study indicated that a PEDV-E variant with a 16–20-aa deletion and an L25P substitution within the transmembrane protein domain upregulated production of an ER stress indicator that enhanced expression of IL-6 and IL-8 and promoted apoptosis *in vitro* (48), while a subsequent reporter assay-based study showed this variant also inhibited promoter activation of type III IFN expression (49). In addition to PEDV-E protein, results of other investigations have suggested that the E protein of Sars-CoV protein may reduce p38 MAPK activation in lung tissues of mice infected with SARS-CoV as compared to those infected with virus lacking E protein (50). Therefore, these data collectively suggest that PEDV-E protein could be a potential transcriptional regulator of expression of certain genes by inhibiting activation of corresponding promoters, in alignment with our results showing that SLA-DR promoters may be regulatory targets of PEDV-E. However, underlying mechanisms whereby virion-associated PEDV-E protein regulates SLA-DRα and β chain gene promoters requires further investigation.

The ORF3 protein, the only PEDV accessory protein with ion channel activity, has been predicted to harbor multiple transmembrane domains and to regulate viral replication and viral virulence (51, 52). Results of previous studies have suggested that PEDV mutants lacking ORF3 expression or that express truncated ORF3 had attenuated virulence phenotypes *in vivo* due to unknown mechanisms (52, 53), implicating involvement of PEDV-ORF3 in viral virulence. Our data suggested that PEDV-ORF3 protein acted as an inhibitor of SLA-DR gene transcriptional activation in cells harboring replicating PEDV (in addition to PEDV-E-induced inhibition), revealing a potential role of PEDV-ORF3 in inhibiting SLA-DR expression during virus replication.

The biological significance of reduced SLA-DR levels in PEDV-infected BM-DCs is still unknown. In our previous report, BM-DC surface SLA-DR heterodimers presented immunepeptides derived from both PRRSV structural and non-structural proteins to CD4+ T cells that could evoke antibody responses *in vivo*; this result was confirmed *via* ELISA detection of antibodies that bound to PRRSV immunepeptides probes from serum samples collected from piglets after PRRSV infection (27). Therefore, impaired SLA-II function should theoretically evoke a delayed antibody response *in vivo*. A previous report suggested that SLA-DR expression in MoDCs after infection with PEDV-CV777 strain for 24 h was increased along with production of higher levels of IL-12 and IFN-γ (13). This result was consistent with results of another report suggesting that an *in vitro* attenuated PEDV strain provided effective immune protection against challenge with virulent PEDV, an effect that was attributed to the ability of the attenuated strain to stimulate BM-DCs (54). Therefore, here we propose that downregulation of expression of cell surface SLA-DR protein molecules on BM-DCs after PEDV infection may have impaired induction of PEDV-specific antibody responses and influenced PEDV virulence. Meanwhile, ORF3 protein also appeared to participate in downregulation of SLA-DR expression at the transcriptional level, but this speculation requires further investigation.

In conclusion, our study demonstrated that PEDV could infect APCs such as DCs to impair expression of SLA-DR, key MHC-II molecules responsible for pathogen antigen presentation in swine. Inhibition of SLA-DR expression mainly occurred at the transcriptional level, not at the protein level, and did not require PEDV replication. Notably, introduction of the envelope protein of PEDV (PEDV-E) into cells could strongly block activation of SLA-DRα and β gene promotors, while introduction of PEDV-ORF3 protein into cells could further inhibit activation of SLA-DRα and β promoters during PEDV replication. Taken together, these results provide novel insights into underlying mechanisms for PEDV pathogenesis *in vivo*.

## Data Availability Statement

The original contributions presented in the study are included in the article/**Supplementary Material**. Further inquiries can be directed to the corresponding authors.

## Ethics Statement

The animal study was reviewed and approved by Animal Welfare Committee of Northwest A&F University.

## Author Contributions

JW, YW, BL, YH, and ZL performed the experiment. YH, ZL, and QZ analyzed the data. YN and CW conceived the study, drafted the manuscript, and finalized the paper. All authors contributed to the revising manuscript.

## Funding

This work was supported by a grant from the National Key Research and Development Program of China awarded to YN (Grant No. 2017YFD0501004).

## Conflict of Interest

The authors declare that the research was conducted in the absence of any commercial or financial relationships that could be construed as a potential conflict of interest.

## Publisher’s Note

All claims expressed in this article are solely those of the authors and do not necessarily represent those of their affiliated organizations, or those of the publisher, the editors and the reviewers. Any product that may be evaluated in this article, or claim that may be made by its manufacturer, is not guaranteed or endorsed by the publisher.

## Supplementary Material

Supplementary Figure 1 Evaluation of serum from recombinant PEDV-N immunized mice. Two weeks after the third immunization of mice with recombinant PEDV-N protein, diluted mice serum was added to plates wells coated with recombinant N protein and incubated for one hour at 37°C. After washing wells with PBS containing 0.5% Triton X-100. Binding of serum polyclonal antibody to N protein was detected by HRP-conjugated goat anti-mouse IgG antibodies followed by visualization using a TMB substrate for absorbance at 450 nm.

Supplementary Figure 2 Infection of Vero cells by PEDV and UV-inactivated PEDV. The Vero cells were infected by Vero cell-adapted PEDV strain KB2013-p120 or UV-inactivated PEDV at 1MOI for 24 hours, then cells were observed under light microscope. Normal cells without PEDV infection were included as control.

Supplementary Figure 3 Expression of PEDV proteins. HEK-293T cells were transfected with plasmids encoding C-MYC tag fused PEDV-S2, M, N, E and ORF3 genes for 48 hours. Next, cells were harvested by SDS-PAGE sample buffers for western blot using anti-C-MYC tag Mab. The 293T cells transfected with empty vector (EV) were included as blank control.

Supplementary Figure 4 Establishment of HEK-293T-SLA-DRα/β. **(A)**. HEK-293T cell were transduced with lentivirus encoding SLA-DRα (zsGreen) and SLA-DRβ (mCherry). The zsGreen and mCherry double positive cells were sorted using flow cytometry and subjected to limited dilution. The HEK-293T-SLA-DRα/β cell subclone 3F3 (live cells) bearing SLA-DRα (zsGreen) and SLA-DRβ (mCherry) were subjected to observation under florescence microscope using FITC channel (Green) and TRITC channel (Red). **(B)** Two HEK-293T-SLA-DRα/β cell subclone 1F9 and 3F3 were subjected to immune precipitation (IP) using Mab recognize assembled whole SLA-DR molecules. Next, the IP complex were subjected to western blot using SLA-DRα Mab and mice serum against recombinant SLA-DRβ. The subclone 3F3 were used in whole study.
