# Supplementary figures and images for: Porcine Epidemic Diarrhea Virus Envelope Protein Blocks SLA-DR Expression in Barrow-Derived Dendritic Cells by Inhibiting Promoters Activation
Source: Front Immunol. 2021 Nov 10;12:741425. doi: 10.3389/fimmu.2021.741425 (PMC8631437; doi:10.3389/fimmu.2021.741425)

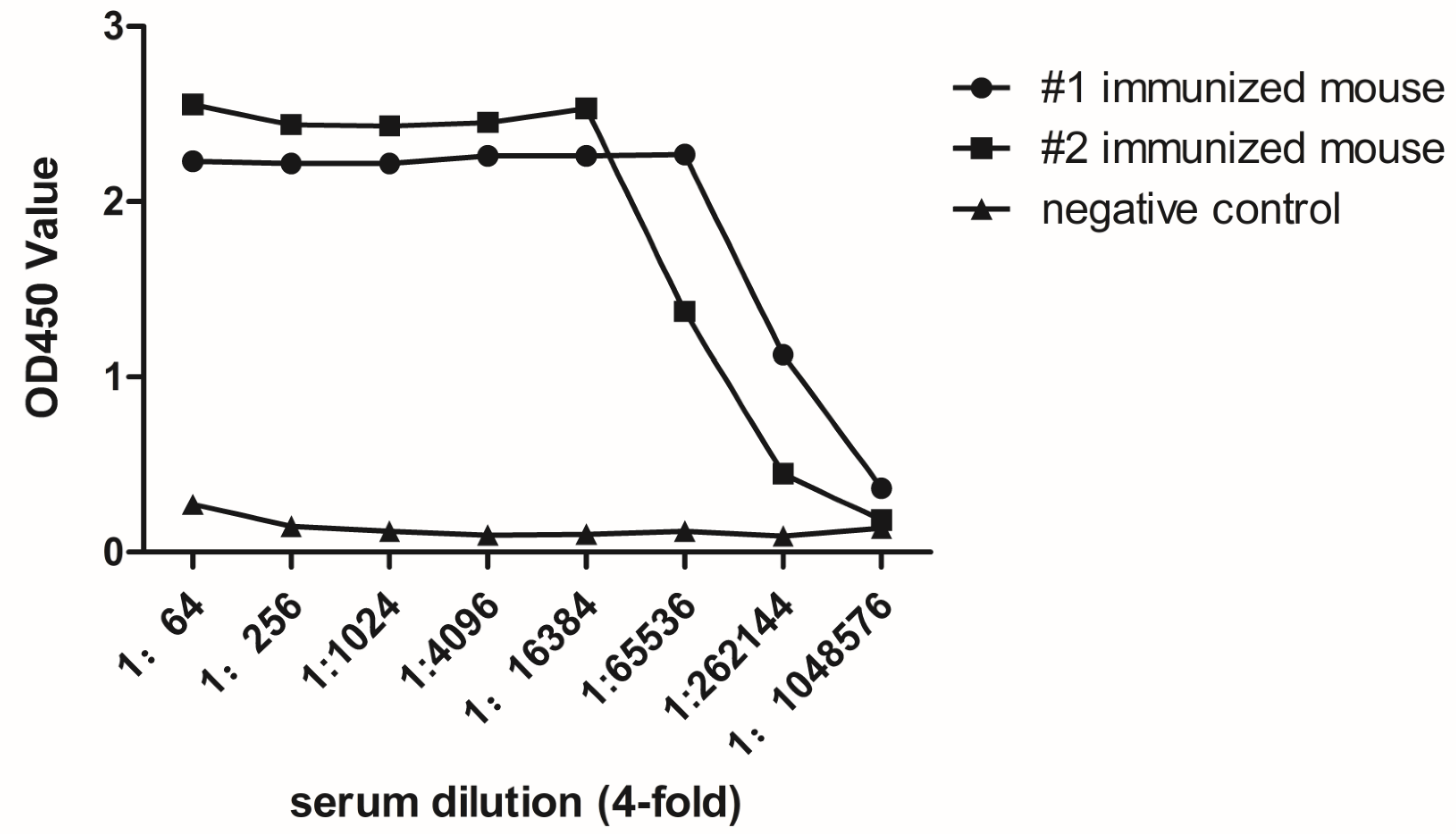

Supplement: Supplementary Figure 1 — Evaluation of serum from recombinant PEDV-N immunized mice. Two weeks after the third immunization of mice with recombinant PEDV-N protein, diluted mice serum was added to plates wells coated with recombinant N protein and incubated for one hour at 37°C. After washing wells with PBS containing 0.5% Triton X-100. Binding of serum polyclonal antibody to N protein was detected by HRP-conjugated goat anti-mouse IgG antibodies followed by visualization using a TMB substrate for absorbance at 450 nm. [file Image_1.tiff]

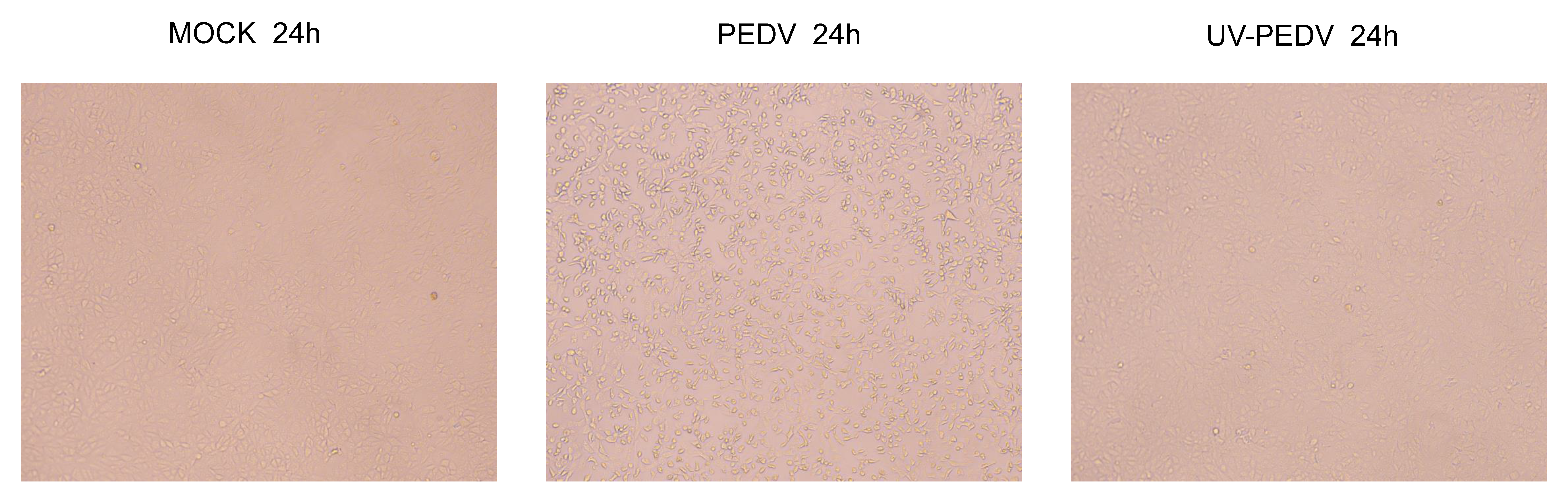

Supplement: Supplementary Figure 2 — Infection of Vero cells by PEDV and UV-inactivated PEDV. The Vero cells were infected by Vero cell-adapted PEDV strain KB2013-p120 or UV-inactivated PEDV at 1MOI for 24 hours, then cells were observed under light microscope. Normal cells without PEDV infection were included as control. [file Image_2.tiff]

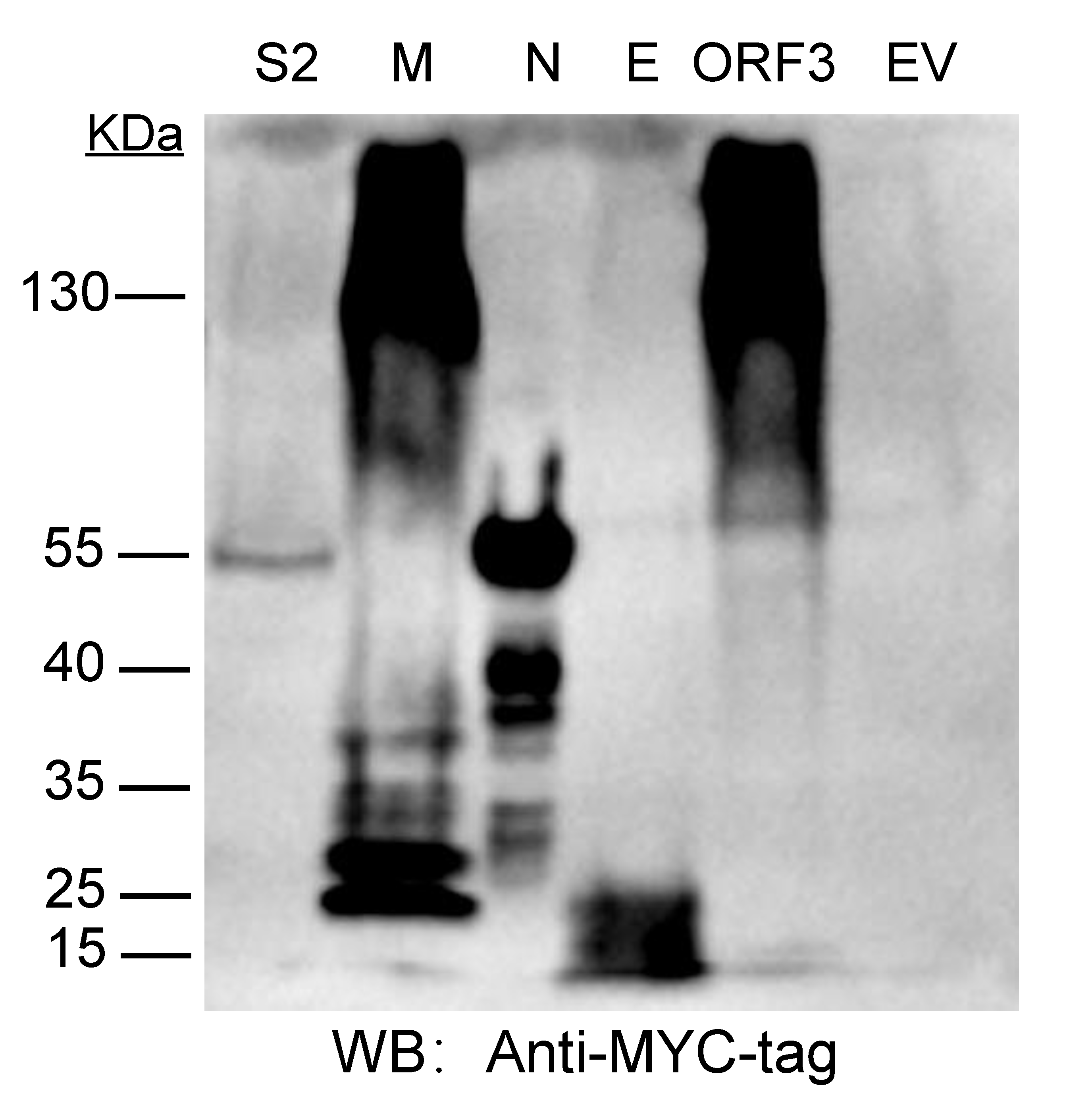

Supplement: Supplementary Figure 3 — Expression of PEDV proteins. HEK-293T cells were transfected with plasmids encoding C-MYC tag fused PEDV-S2, M, N, E and ORF3 genes for 48 hours. Next, cells were harvested by SDS-PAGE sample buffers for western blot using anti-C-MYC tag Mab. The 293T cells transfected with empty vector (EV) were included as blank control. [file Image_3.tiff]

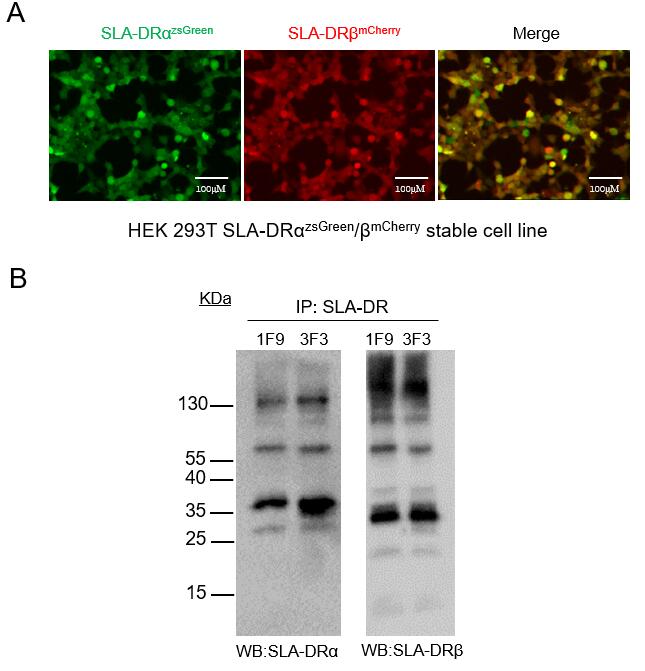

Supplement: Supplementary Figure 4 — Establishment of HEK-293T-SLA-DRα/β. (A). HEK-293T cell were transduced with lentivirus encoding SLA-DRα (zsGreen) and SLA-DRβ (mCherry). The zsGreen and mCherry double positive cells were sorted using flow cytometry and subjected to limited dilution. The HEK-293T-SLA-DRα/β cell subclone 3F3 (live cells) bearing SLA-DRα (zsGreen) and SLA-DRβ (mCherry) were subjected to observation under florescence microscope using FITC channel (Green) and TRITC channel (Red). (B) Two HEK-293T-SLA-DRα/β cell subclone 1F9 and 3F3 were subjected to immune precipitation (IP) using Mab recognize assembled whole SLA-DR molecules. Next, the IP complex were subjected to western blot using SLA-DRα Mab and mice serum against recombinant SLA-DRβ. The subclone 3F3 were used in whole study. [file Image_4.jpg]
